# The study of multisensory interception for interaction with objects and others in visually impaired children

**DOI:** 10.3389/fnhum.2025.1645731

**Published:** 2025-08-11

**Authors:** Tommaso Bartolini, Martina Riberto, Helene Vitali, Mark T. Wallace, Monica Gori

**Affiliations:** ^1^Unit for Visually Impaired People, Italian Institute of Technology, Genoa, Italy; ^2^DIBRIS, University of Genova, Genoa, Italy; ^3^Department of Psychology, Vanderbilt University, Nashville, TN, United States

**Keywords:** interception, multisensory integration, social interaction, motor development, visual impairment, VI children

## Abstract

Interception refers to goal-directed motor actions aimed at interacting with moving objects and is essential for both motor co-ordination and social engagement. In childhood, interceptive skills support environmental exploration, peer interaction, and participation in play and sports. For children with visual impairments, the lack of visual cues compromises the development of these skills, potentially limiting motor competence and opportunities for social interaction. Despite its clinical and developmental relevance, research on interception in visually impaired (VI) children is extremely limited. This mini review synthesizes findings from studies on interceptive skills in VI adults, as well as in sighted and VI children. We discuss how vision contributes to interceptive actions, and how alternative sensory pathways can compensate in its absence. We highlight major limitations of current literature, including poor ecological validity, a lack of longitudinal data, and scarce attention to multisensory and social aspects. To address these gaps, we propose future research directions that include cross-sectional and longitudinal studies, multisensory paradigms, and the use of virtual reality technologies to simulate naturalistic environments. These approaches may inform inclusive and rehabilitative interventions that support the motor and social development of VI children through accessible, engaging, and developmentally appropriate interceptive experiences.

## Introduction

The term interception refers to any goal-oriented motor action to interact with a moving object, such as attempting to catch, hit, or stop it (Brenner and Smeets, 2018). Interceptive actions are required in daily life for numerous activities (e.g., handling objects in motion, sports, games) and enable crucial survival behaviors, such as catching prey and avoiding potentially harmful collisions. Successful interception relies on different skills, including spatial orientation, multisensory integration and motor coordination (Bahadori et al., 2023; Zago et al., 2009). Vision is the primary sense involved in interception tasks, which require the ability to predict an object’s trajectory, speed, and timing. Hence, vision provides essential sensory input for accurate motor coordination, spatial awareness, and interaction with the surrounding (Brian et al., 2021). The ability to process visual information rapidly and accurately allows individuals to determine when and where a collision with an object will occur, thereby facilitating spatially and temporally precise motor responses (Brenner and Smeets, 2018).

Consequently, visually impaired (VI) individuals often face significant challenges in executing interceptive actions, because they lack the real-time visual cues that typically guide moving objects tracking and motor coordination. The difficulties in interception tasks may differ according to the severity of the visual impairments, ranging from blindness to low vision. This phenotype may depend on the etiology (e.g., optic nerve hypoplasia, cortical visual impairment), timing of onset (e.g., congenital versus later-acquired), residual vision, and the presence of additional neurodevelopmental or systemic conditions (e.g., cerebral palsy, intellectual disability) (Solebo and Rahi, 2014). All these aspects critically influence developmental trajectories and support needs. In particular, absent or reduced visual input at early life stages can lead to delayed or impaired motor development, with detrimental effects on social interactions and interactions with the environment (Fotiadou et al., 2014). This is because the development of both gross and fine motor skills allows children to actively engage with their environment through movement and object manipulation. Even simple actions, such as reaching for and manipulating an object, require a complex integration of multisensory and motor information. This process is especially critical in VI children, where the integration between perception and action is deeply compromised (Gori et al., 2025). Research has shown that children with more advanced motor skills are more likely to develop stronger and more complex social relationships, as their ability to move and interact with their environment facilitates communication, cooperation, and shared activities with others (Herrmann et al., 2021). However, in the presence of congenital or early acquired visual impairment, this natural developmental cascade, from motor competence to social engagement, becomes significantly disrupted. This impairment can manifest as difficulties in spatial orientation, balance, movement execution, and consequently less opportunities for social participation. Over time, this may lead to social isolation and increase the risk of psychological difficulties, including low self-esteem and feelings of exclusion (Ozaydin, 2015). Among the various types of movement that facilitate social interaction in childhood, interceptive actions play a crucial role, particularly in the context of games and sports (Buszard et al., 2021). Therefore, it is essential to study the development of this type of movement in relation to the progression of motor and social skills in absence of visual input. This is particularly important during infancy as one of the critical periods with heightened plasticity in sensory and sensorimotor areas (Larsen et al., 2023). Initiating targeted motor activities during this period is crucial for VI children to support adequate sensorimotor integration and to foster compensatory strategies through non-visual sensory pathways.

Below, we review the major findings in the literature on interception with the goal of placing this concept within the context of a multidisciplinary research. With this aim, we will summarize two lines of research (Table 1), one on adults and the other on children, with particular focus on clinical populations with congenital or early acquired visual impairments. We will discuss sensorimotor and social aspects of interception, methodological limitations, and propose future directions based on ecological paradigms and rehabilitation applications.

**TABLE 1:** Summary of the studies on interception, in adults and children, with typical and atypical visual development.

Tasks	VI adults	Typical children	VI children
Real object interception	Performance comparable to blindfolded sighted adults (Vernat and Gordon, 2011, 2010).	Interceptive skills (e.g., catching or hitting moving objects) improve with age between 5 and 12 years old (Chohan et al., 2008). Older children (10–12 years) show adult-like performance in standard tasks (Chohan et al., 2008). In more challenging conditions (i.e., when the object’s trajectory is temporarily occluded), absence of age-related differences in interception accuracy (van Kampen et al., 2010).	Missing
Virtual object interception	Missing	Accuracy in intercepting moving targets increases with age (Rothenberg-Cunningham and Newell, 2013). Younger children (7–8 years) showed slower movements velocities and longer response times than older participants (Rothenberg-Cunningham and Newell, 2013). Positive correlation between interceptive-timing ability and mathematics performance in children aged 5–11 years (Giles et al., 2018). In children aged 9–11 years, external focus (on the environment or task outcome) led to greater interception accuracy than internal focus (on body movements) (Abdollahipour et al., 2023).	Missing

## Interception in the absence of visual input

As previously mentioned, vision is the main sensory input to perform successful interceptive actions, although visual input is not strictly necessary for effective interception. Indeed, animals rely on various sensory modalities (e.g., olfaction) to capture prey and navigate their environment (Johnson et al., 2004; Surlykke and Kalko, 2008; Tricas, 1982). Similarly, in absence of visual information, humans can intercept moving targets using temporal cues (e.g., time of release), intuitive knowledge of falling object dynamics (e.g., height, mass) and tactile information (Lacquaniti and Maioli, 1989; Nelson et al., 2019). Also, in auditory interception tasks, the accuracy in predicting the time of impact of a virtual ball represented as a moving sound increased with the sound intensity (Tolentino-Castro et al., 2024). Interestingly, these findings support the idea of a shared anticipation mechanisms among different sensory modalities, wherein successful interception is predicted by the perceived position and velocity of moving stimuli (Brenner and Smeets, 2015).

In line with this, very few studies have explored interception in VI adults in both experimental and real-world contexts. Those that have been done demonstrated the ability to successfully intercept a target by using auditory spatial and temporal cues to coordinate their actions (Vernat and Gordon, 2011, 2010). Specifically, VI participants were asked to intercept a ball rolling down a track by launching another ball along a perpendicular path. They could estimate its position and speed relying solely on the sound produced by the moving ball. Their performance was comparable to that of blindfolded sighted adults, suggesting similar mechanisms underlying interceptive abilities in the absence of vision, regardless of whether this absence is congenital or experimentally induced.

In more ecological contexts, the growing popularity of sports based on ball interception designed for VI athletes (e.g., based on audio feedback) clearly demonstrate the possibility to develop fine interceptive skills even in individuals with degraded or completely absent visual input. Games such as showdown (Kokhan et al., 2020), blind football (Velten et al., 2016) and sonic badminton (Kim et al., 2016) include environmental and technical features that amplify auditory feedback from the ball, enabling VI players to perform precise interceptive actions. Indeed, research shows that, in a ball-passing task, amateur VI football players kick the ball with a foot speed similar to that of sighted players and use specific motor strategies to maximize postural stability and navigate space safely (Finocchietti et al., 2019). Although VI individuals could develop motor skills and engage in meaningful social interactions, these sports typically require specialized settings, equipment, and trained partners. This limits the opportunities for independent or home-based practice, highlighting the need for more accessible devices or games that can support the development of interceptive abilities in everyday life.

## Typical and atypical development of interceptive skills

In the previous section, we reviewed studies in VI adults, showing that they can develop fine interception skills even in absence of visual input. However, acquiring these skills can be especially challenging, particularly when the visual impairment is present from birth or develops early in life. While several studies have investigated interception skills in developmental disorders, such as autism spectrum disorder and motor coordination disorder (Adams et al., 2014; Park et al., 2024), to our knowledge, no research has yet to explore the development of interception skills in VI children. To date, most of the studies in VI children in this field of research have focused on the delay in the development of both fine and gross motor skills to grasp objects compared to their sighted peers (Brian et al., 2021; Ebrahimi et al., 2024; Houwen et al., 2007; Widyawan, 2021).

Although limited, the development of interceptive skills has been explored in sighted children using both real-object interception and virtual interception tasks, with mixed findings. On the one hand, work has found an effect of age on the development of interceptive skills, with older children (10–12 years) being able to adapt their movements and adjust their locomotor behavior (e.g., walking speed) to reach and grasp a moving objects like adults (Chohan et al., 2008). These results pointed at an underdeveloped coupling between self and object motion in young children (5–7 years). In line with these findings, one study examined age-related differences across various age groups (i.e., 7–8, 11–12, 15–16, and 19–20 years old) in a virtual interception task. They observed that the accuracy in interceptions increased with age, with younger children exhibiting slower velocities and longer response latencies in catching the moving visual target compared to older groups (Rothenberg-Cunningham and Newell, 2013). In contrast, in more difficult task conditions (when the object’s trajectory was temporarily occluded), no significant age-related difference were seen in interception accuracy (van Kampen et al., 2010).

Another line of research explored the relationship between motor timing skills and academic achievement, specifically in mathematics, in children aged 5–11. Participants performed a virtual interception task; specifically, the interception of a moving target with curvilinear trajectories displayed on a screen using a cursor. The goal of this study was to test whether the timing in intercepting the target with the cursor was correlated with mathematical skills. They found a positive correlation between interceptive-timing ability and mathematics attainment (Giles et al., 2018). This study aligns with a broader line of research suggesting that early acquisition of sensorimotor skills contributes to the formation of numerical representations (Nieder and Dehaene, 2009).

Finally, another study found that attentional focus might be related to motor performance and interception accuracy. In this task, children aged 9–11 were instructed to hit a tennis ball with a stick and divided into three experimental groups according to the attentional focus: external (i.e., focus on the blade of the stick), internal (i.e., focus on your hands) and a control condition without any specific focus instructions. They observed higher accuracy in the external focus condition compared to the internal focus condition (Abdollahipour et al., 2023). This result could be interpreted according to the constrained action hypothesis, which posits that directing attention internally (e.g., on body movements) interrupts automatic motor control processes and subtracts attentional resources, thus disrupting motor performance. In contrast, an external attention focus (e.g., on a ball or target) should foster automatic motor control and enhance motor performance (Wulf et al., 2001). Interestingly, the possibility of directing attentional focus to improve motor performance in VI individuals has not been explored. Future studies should investigate whether external focus is effective for them, or if an internal focus might be more beneficial in absence of visual input.

Major findings in research on interception are summarized in Table 1. Overall, the studies in children addressed different aspects of interceptive abilities, including motor control and attention. The amount of work in children is quite limited when compared with adults and includes some limitations that we will discuss below.

## Limitations in the interception literature

Despite the relevance of interception in development and from a clinical perspective, previous research presents several methodological and theoretical limitations. First, the study of interception requires the integration of multiple types of data, including psychophysical measurements, kinematic analysis of subjects’ movements, and motion tracking of object trajectory. In addition, designing experiments with high ecological validity is particularly difficult, as it requires controlling multiple variables while ensuring that tasks reflect real-world scenarios. Traditional experimental setups often fall short in replicating the complexity of natural interception, limiting the generalizability of findings. Some studies employed virtual interception tasks (e.g., Rothenberg-Cunningham and Newell, 2013), while others used unnatural real-interception setups, such as intercepting objects moving along a fixed track (e.g., Chohan et al., 2008; van Kampen et al., 2010). Although these paradigms are valuable for examining specific aspects of interceptive behavior, such as motor timing and trajectory prediction, they do so at the cost of failing to capture the richness of real-world interceptive actions.

Beyond these methodological challenges, the developmental trajectories of interception skills remain poorly understood. Behavioral data indicated an age-related effect on interception accuracy (Chohan et al., 2008; Rothenberg-Cunningham and Newell, 2013), possibly due to a limited ability to predict the target’s trajectory and to adjust movements accordingly. However, most of the studies did not explore critical components for the development of effective interception, such as sensory integration and real-time sensorimotor coordination. In addition, few studies have been conducted in the VI population, with only two studies in blind adults (Vernat and Gordon, 2011, 2010). To better understand these crucial aspects of the development of interceptive skills, it is necessary to design both longitudinal and cross-sectional studies in naturalistic settings, involving samples of both sighted and VI children.

Moreover, to our knowledge, no studies have investigated the neural correlates of interceptive action in children. Neuroimaging studies in sighted adults have shown the recruitment of the dorsal visuomotor pathways and posterior parietal regions during transversal motion interception (Senot et al., 2008), and the involvement of the anterior cingulate cortex for attentional control during interception tasks (Tombini et al., 2009). The developmental trajectories of these pathways in sighted children, and whether they differ in VI children remain open questions. This represents a significant gap in the literature to understand their strategies to compensate for the partial or total lack of visual input and to plan effective clinical interventions.

Finally, social interception, namely the interception in social interactions, for example with peers, remains largely unexplored. Interception often occurs in rich interactive contexts, such as group games, sports, or dyadic play, where children need to coordinate actions with one another, manage conflicting intentions, and adapt dynamically in real time. Also, the affective dimensions of the physical contact (e.g., hand-clapping games) along with the collaborative/competitive behaviors play a critical role in children’s sensorimotor development, social cognition, and ability to engage in coordinated, goal-directed group activities. Yet, much of the existing research focused on tasks that did not require any interaction or collaboration between participants as takes place in real-world contexts (e.g., ball games).

## Future perspectives

To fill these gaps, the study of interceptive skills in childhood will require methodological innovations and a more appropriate ecological framework and carry out this work in a longitudinal fashion to monitor the development of the child with and without visual impairment. A promising advancement for the study of interception could be the integration of virtual reality (VR) environments with spatiotemporal tracking systems. VR can simulate dynamic and interactive scenarios, allowing children to engage in more naturalistic interception tasks, while tracking technologies can precisely measure reaction times, motor coordination, and spatial awareness. Furthermore, VR environments enable precise control over the perceptual characteristics of stimuli (e.g., duration, trajectory, sensory modality), allowing for accurate psychophysical measurements without compromising the ecological validity of the setting. This combined approach would enhance both the ecological validity and the precision of experimental studies on interception skills. Finally, VR technologies could be used to create virtual scenarios in which VI children practice interceptive tasks through non-visual cues. Building on previous work (Kammoun et al., 2012; Lahav and Mioduser, 2004), spatialized audio can guide children to localize moving targets by sound alone, while haptic devices can signal the optimal timing or direction for interception.

Also, although existing studies indicated improvements in motor accuracy and prediction abilities with age, little is known about how perceptual, cognitive, and motor systems interact over time to support this progression. Longitudinal and cross-sectional studies could help in identifying crucial developmental stages of interception skills, both in sighted and in VI children. Investigating how VI children interact with moving stimuli can reveal important compensatory strategies to plan training protocols based on alternative sensory pathways. One promising approach is combining multisensory integration paradigms with interception tasks that involve motor control and social engagement (e.g., games and sports). Multisensory integration has been shown to enhance perception and interaction with the environment in VI individuals (Gori, 2015), suggesting that similar benefits might extend to interceptive actions. Moreover, early visual deprivation triggers cross-modal plasticity, leading to a functional reorganization of the occipital cortex that supports the processing of non-visual inputs (Collignon et al., 2013). Combining multisensory paradigms with appropriate motor training may therefore foster the development of non-visual interceptive skills, allowing VI children to effectively anticipate and interact with moving stimuli through auditory and tactile cues.

Finally, future research should focus on the social dimension of interception for social bonding, communication, and inclusion of VI children. Research on joint action shows that perceiving others’ actions activates our motor systems as if we were preparing the actions ourselves, facilitating action coordination and execution (van der Wel et al., 2021). Interactive paradigms, like the doubles-pong task (Benerink et al., 2016), offer a useful model for studying these processes and could also be extended to examine how VI children engage in non-verbal coordination with sighted and other VI peers. Beyond competitive/cooperative games, interception is also part of affective interactions, such as hand-clapping, which require precise spatial and temporal alignment and support both motor development and social connectedness. Studying these aspects of social motor interaction may provide valuable insights into imitation and turn-taking in joint action contexts (McEllin et al., 2018). A further promising direction involves examining how children respond to different types of agents during interception, that is, whether a ball is thrown by another child, a robot, or a simulated human in VR. Such comparisons may reveal how children distinguish between living and non-living objects, attribute intentionality, and modulate their behavior accordingly. Investigating these distinctions might be relevant for social cognition in action and inform the design of inclusive, interactive learning and rehabilitation environments.

In conclusion, the study of multisensory interception requires a multifaceted approach that integrates psychophysics, kinematic analysis, VR, spatiotemporal tracking technologies, and brain imaging to understand how interception skills develop in both sighted and VI children. Considering the interplay between motor and social skills (Leonard and Hill, 2014), the improvement in interception abilities could influence not only motor coordination, but also facilitate peer engagement, reducing social isolation and promoting psychological well-being. The results from this new line of research could have implications for the development of innovative rehabilitation and inclusive strategies for motor and social development in VI children.
